# Structural and functional analysis of *Hydra* Actinoporin-Like Toxin 1 (HALT-1)

**DOI:** 10.1038/s41598-021-99879-5

**Published:** 2021-10-19

**Authors:** De-Sheng Ker, Hong Xi Sha, Mohd Anuar Jonet, Jung Shan Hwang, Chyan Leong Ng

**Affiliations:** 1 Institute of Systems Biology, Universiti Kebangsaan Malaysia, 43600 UKM Bangi, Selangor Malaysia; 2 Department of Biological Sciences, School of Medical and Life Sciences, Sunway University, No. 5, Jalan Universiti, 47500 Bandar Sunway, Selangor Malaysia; 3 Malaysia Genome Institute, National Institutes of Biotechnology Malaysia, Jalan Bangi, 43000 Kajang, Selangor Malaysia; 4 Department of Medical Sciences, School of Medical and Life Sciences, Sunway University, No. 5, Jalan Universiti, 47500 Bandar Sunway, Selangor Malaysia; 5 Present Address: York Structural Biology Laboratory, Department of Chemistry, University of York, York, YO10 5DD UK

**Keywords:** Molecular biology, Structural biology

## Abstract

Actinoporins are a family of α-pore-forming toxins (α-PFTs) that have been identified in sea anemones. Recently, a freshwater *Hydra* Actinoporin-Like Toxin (HALT) gene family was found in *Hydra magnipapillata*. Unlike sea anemone actinoporins that use sphingomyelin as their main recognition target, the HALTs proteins may recognise alternative lipid molecules as their target. To unveil the structural insights into lipid preference of HALTs protein as compared to sea anemone actinoporins, we have determined the first crystal structure of actinoporin-like toxin, HALT-1 at 1.43 Å resolution with an acetylated lysine residue K76. Despite the overall structure of HALT-1 sharing a high structural similarity to sea anemone actinoporins, the atomic resolution structure revealed several unique structural features of HALT-1 that may influence the lipid preference and oligomerisation interface. The HALT-1 contains a RAG motif in place of the highly conserved RGD motif found in sea anemone actinoporins. The RAG motif contributed to a sharper β9-β10 turn, which may sway its oligomerisation interface in comparison to sea anemone actinoporins. In the lipid-binding region, the HALT-1 contains a shorter α2 helix and a longer α2-β9 loop due to deletion and subsequently an insertion of five amino acid residues in comparison to the sea anemone actinoporins. Structure comparison and molecular docking analysis further revealed that the HALT-1 lipid-binding site may favour sphingolipids with sulfate or phosphate head group more than the sphingomyelin. The structure of HALT-1 reported here provides a new insight for a better understanding of the evolution and lipid recognition mechanism of actinoporin.

## Introduction

Pore-forming toxins (PFTs) are commonly found in pathogenic cell invasion of bacteria as well as in the offensive and defensive mechanism of eukaryotes^1^. These toxins form oligomeric pores in the biological membrane, leading to the influx of ions and water that subsequently causing colloid-osmotic lysis of cells^2^. PFTs appear ubiquitously throughout the life cycle of prokaryotes and eukaryotes, and they are extremely structurally diverse. As compared to their bacterial counterparts, eukaryotic PFTs are less well studied, and it was until recently that much attention was given to understand the structural and functional diversity of eukaryotic PFTs, likely due to their potential in the biotechnological applications^3^. Depending on whether the transmembrane region is made up of α-helices or β-strands, PFTs can be grouped into α-PFTs and β-PFTs. Amongst these PFTs are the actinoporins, a large family of α-PFTs that are mainly found in sea anemones (Phylum Cnidaria, Class Anthozoa). They have an average size range from 17 to 20 kDa with their monomeric structure having an anti-parallel β-sheet flanked by two α-helices.

To date, all reported actinoporin structures are found in marine sea anemones including equinatoxin II from *Actinia equina* (AeEqt-II)^4^, sticholysin II from *Stichodactyla helianthus* (ShSt-II)^5,6^, fragaceatoxin C from *Actinia fragacea* (AfFraC)^7^, and fragaceatoxin E from *Actinia fragacea* (AfFraE)^8^. Individual actinoporins can oligomerise and form a toroidal pore with a diameter of 2 nm^9^. The classical route of pore formation is initiated by the contact of actinoporin with the membrane sphingomyelin, followed by a conformational change and assembly of individual actinoporins into the transmembrane oligomer with a central lumen^10^. When monomeric actinoporins approach the surface of cell membranes, they recognise and bind specifically to sphingomyelin^10,11^. Sphingomyelin is the only membrane target recognised by all actinoporins that had been identified so far from sea anemones. Others such as cholesterol, phosphatidylcholine, and carbohydrate have also been reported as the essential membrane components for facilitating the actinoporin binding^12–14^. The binding to cell membranes is conducted via the cluster of aromatic residues exposed on the protein surface. These aromatic residues are positioned in the loop region between β-strand 6 (β_6_) and 7 (β_7_) as well as in α-helix 2 (α_2_) and the loop following α_2_^15^. The N-terminal α-helix of actinoporins is amphipathic, and it integrates into the cell membrane to form a stable protein-lipid interface supported by polar and non-polar interactions^16–18^. Upon forming discrete pores in the cell membrane, monomeric actinoporins self-assemble via an intermediate transition of dimeric state and eventually form either tetramer, hexamer, or octamer with the central ionic lumen that allows small ions (Ca^2+^, K^+^, Na^+^, and Cl^−^) to pass through the membrane barrier^19,20^.

*Hydra* is a genus of freshwater cnidarians that can be found in lakes, ponds, and rivers. Small crustaceans and fish larvae are common preys for *Hydra* and when they come in contact with *Hydra* venom, they would be instantly killed or paralysed^21^. The *Hydra* venom was extracted and tested directly on *Drosophila* and human erythrocytes, and the results clearly showed that the haemolytic activity of *Hydra* venom was both dose- and time-dependent^22^. Recently, a *Hydra* Actinoporin-Like Toxin (HALT) gene family has been found in *Hydra magnipapillata* and composed of seven paralogs, in which HALT-1, HALT-4, and HALT-7 are secreted as part of venom by *Hydra* during its prey hunting^23,24^. The protein was named as “actinoporin-like toxin” because of its protein sequence similarity (~ 30%) with actinoporins^23^. The HALT-1 has been predicted to have the amphipathicity of N-terminal α-helix and the clusters of aromatic residues similar to other actinoporins with different membrane target site(s)^23^. Instead of sphingomyelin, HALT-1 is likely to bind to sulfatide, a sphingolipid with sulfate group attached to 3′ carbon of the galactose group^24^. In addition, the HALT-1 has been demonstrated to have a binding specificity toward lysophosphatidic acid (LPA) and sphingosine-1-phosphate (S1P)^24^. The recombinant HALT-1 was found to exert haemolytic activity, and also capable to cause physical lysis on the majority of human cell lines^23,25,26^.

Hereby, we report the first crystal structure of the HALT gene family. The structure of HALT-1 was compared with available structures of sea anemone actinoporins and provided new structural insight into the binding specificity of actinoporins.

## Results and discussion

### Multiple sequence alignment (MSA) of HALTs and sea anemone actinoporins

**Figure 1:**
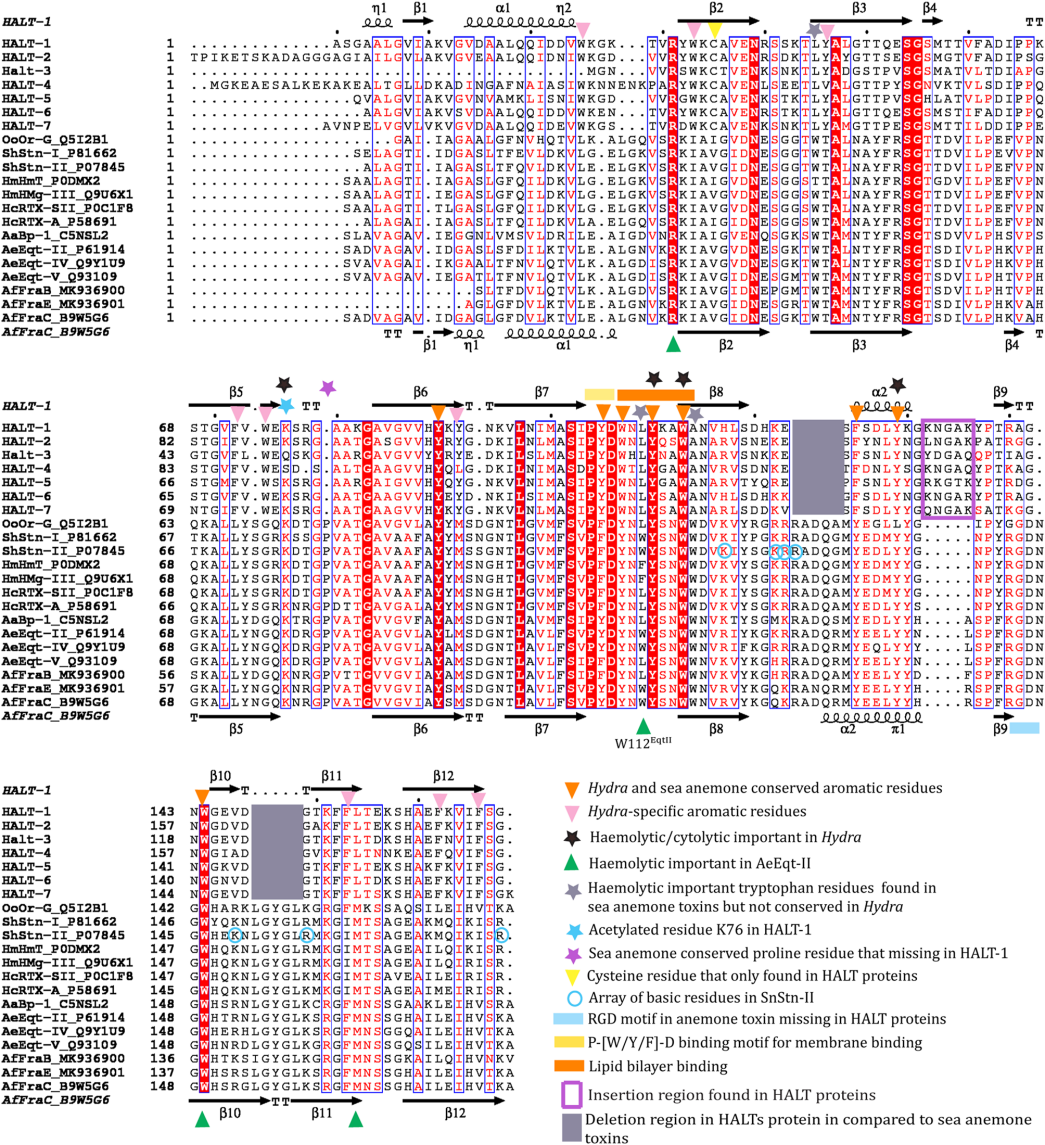
Multiple sequence alignment (MSA) of HALT proteins with actinoporin homologs from sea anemones. The naming system for the sequences is as follows: Actinoporin-name_Uniprot number. OoOr-G_Q5I2B1: DELTA-actitoxin-Oor1b from *Oulactis orientalis*; ShStn-I_P81662: DELTA-stichotoxin-She4a from *Stichodactyla helianthus*; ShStn-II_P07845: DELTA-stichotoxin-She4b from *S. helianthus*; HmHmT_P0DMX2—DELTA-stichotoxin-Hmg2b from *Heteractis magnific*; HmHMg-III_Q9U6X1: DELTA-stichotoxin-Hmg2a from *H. magnific*; HcRTX-SII_P0C1F8: DELTA-stichotoxin-Hcr4b from *Heteractis crispa*; HcRTX-A_P58691: DELTA-stichotoxin-Hcr4a from *H. crispa*; AaBp-1_C5NSL2: DELTA-actitoxin-Aas1a from *Anthopleura asiatica*; AeEqt-II_P61914: DELTA-actitoxin-Aeq1a from *Actinia equina*; AeEqt-IV_Q9Y1U9: DELTA-actitoxin-Aeq1c from *A. equina*; AeEqt-V_Q93109: DELTA-actitoxin-Aeq1b from *A. equina*; AfFraB_MK936900: fragaceatoxin B from *Actinia fragacea*; AfFraE_MK936901: fragaceatoxin E from *A. fragacea*; AfFraC_B9W5G6: fragaceatoxin C from *A. fragacea* and HALT1-HALT7: HALT proteins. The MSA was carried out using Clustal Omega and the secondary structure of HALT-1 and AfFraC were incorporated using ESPript 3.0^29^.

*Hydra magnipapillata* contains seven members of the actinoporin-like toxins (HALTs) protein family with all of them originating from a common ancestor with a sequence identity of 55–88% among themselves. The HALTs proteins only share 23–30% sequence identity to sea anemone actinoporins including AeEqt-II, ShStn-II, AfFraC, and AfFraE^23,24^. Nonetheless, conserved residues and motifs that were found in sea anemone actinoporins were also present in HALTs including an aromatic residues cluster P^104^-[W/Y/F]-D^106^, a motif that interacts with lipid molecule of the membrane (Fig. 1)^7,23^. In line with the low sequence identity to actinoporin homolog, the HALT protein family was found to diverge through deletions and insertions (Fig. 1). The RGD motif that is involved in oligomerisation interaction of actinoporin, is not conserved in HALT protein family^27^. Moreover, the aromatic residue of tryptophans that function in haemolytic activity and sphingomyelin membrane binding in actinoporins are substituted with non-aromatic residues L45, L109, and A114 in HALTs^7,28^. Despite HALTs exhibit haemolytic and cytolytic activities that are similar to actinoporins, HALTs were recently shown to bind to sulfatide (SFT) as the primary target, unlike AfFraC, AeEqt, and ShStn-II that bind predominantly to sphingomyelin. In addition, HALT-1 and HALT-3 could bind to lysophosphatidic acid (LPA) and sphingosine-1-phosphate (S1P)^24^, suggesting that the overall low sequence identity of HALTs to actinoporin might have contributed to the substrate specificity upon evolutionary events over time.

### Recombinant *Hydra* Actinoporin-Like Toxin 1 (rHALT-1) is a monomer in the solution

The rHALT-1 was highly expressed and soluble in *E. coli*, and high purity of rHALT-1 was obtained after two rounds of purification, which were Ni–NTA affinity chromatography followed by size exclusion chromatography (SEC). The presence of a single peak (~ 15 kDa) in the elution volume of SEC indicated that rHALT-1 existed as a monomer in the solution (Supplementary Fig. S2). The rHALT-1 with a calculated molecular weight of 20 kDa and the elution at a retention volume of ~ 15 kDa indicated that there was a delay during the elution in SEC. This was possibly due to its non-specific interaction with the column. Similarly, a previous study of AfFraC encountered a substantial delay during the SEC elution and suggested that the delay was caused by the interaction of AfFraC with the agarose-dextran matrix of Superdex column^14^.

### Structure of recombinant *Hydra* Actinoporin-Like Toxin 1 (rHALT-1)

To further understand the function and structure of HALTs, we determined the crystal structure of HALT-1 that represents the first atomic structure for HALTs family proteins that only share ~ 30% sequence identity to sea anemone actinoporins. The rHALT-1 consists of 168 amino acid residues with 18 of its signal peptide residues removed. It was cloned and purified using a 6x-His-tag fusion at the N-terminus (Supplementary Fig. S1). A crystal structure of rHALT-1 (PDB ID: 7EKZ) was determined to 1.43 Å resolution. The crystal belonged to a space group P212121 with one monomer in the asymmetric unit (Table 1). The final model of rHALT-1 was composed of 166 residues. The electron density for the first 23 amino acids at the N-terminal including His-affinity-tag was not seen. The amino acid numbering for the HALT-1 in this study started at the amino acid immediately after the signal peptide which had been omitted in rHALT-1 (Supplementary Fig. S1).

**Table 1:** Crystallographic data.

	rHALT-1
Wavelength (Å)	1.54187
Resolution range (Å)	23.55–1.43 (1.45–1.43)
Space group	P2_1_2_1_2_1_
**Unit cell**	
a, b, c (Å)	47.10, 49.59, 77.09
α, β, γ (°)	90.0, 90.0, 90.0
Measured reflections	158,924
Unique reflections	33,402 (1577)
R_sym_	0.147 (0.419)
CC (1/2)	0.983 (0.774)
Mean I/σI (I)	5.2 (2.0)
Multiplicity	4.8 (3.0)
Mosaicity (°)	0.94
Completeness (%)	98.2 (95.2)
**Refinement statistics**	
R_cryst_, R_free_ (%)	16.3, 22.0
No. of molecules per asymmetric unit	1
No. of water molecules	345
No. of glycerol molecules	3
No. of formic acid molecules	3
Root mean square deviation from ideal values (r.m.s.d.)	
Bond length (Å)	0.021
Bond angle (°)	2.11
**Ramachandran plot statistics**	
Favoured regions (%)	98.1
Allowed regions (%)	1.9
Average B factors (all atoms)	13.0
PDB code	7EKZ

Values for the outer shell are given in parentheses.

**Figure 2:**
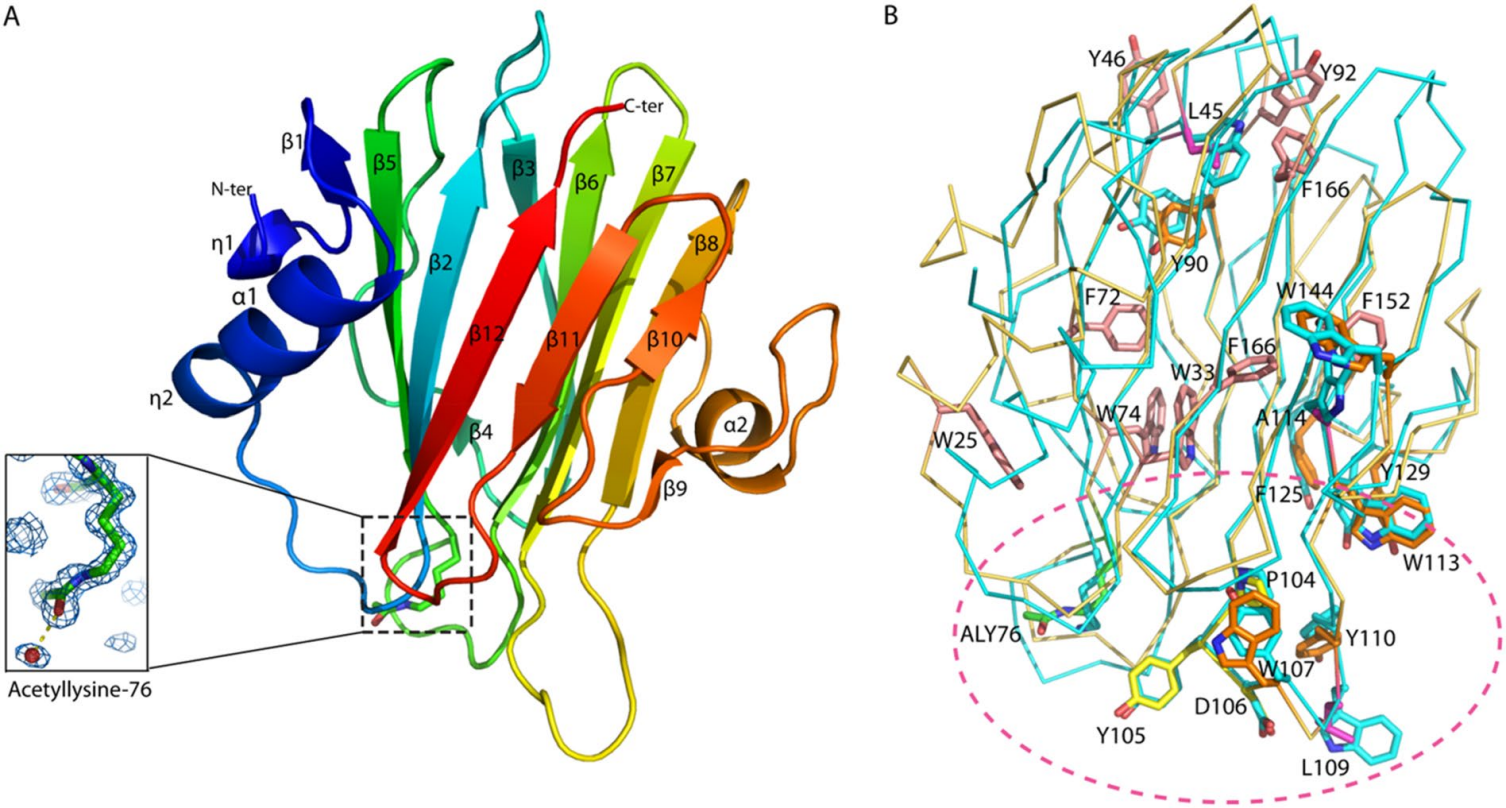
Overall crystal structure of recombinant HALT-1 protein. (**A**) The rHALT-1 consists of β sandwich fold with 12 β-strands and two α-helices and two 3_10_ helices on the sides, which assembles a pore-forming toxin (PFT). The 2Fo-Fc maps for acetylated lysine residue 76 are contoured at 1.0 σ. (**B**) Superimpose of HALT- 1 (brown) and AfFraC (PDB ID: 4TSQ_A) (cyan). The aromatic residues that are found conserved in HALT proteins are shown in light pink stick and aromatic residues that are found conserved in both *Hydra* and sea anemone toxins are shown in orange stick. Residues L45, L109, and A114 in HALTs are shown in pink stick. They are variants to aromatic residues found in sea anemone toxins that are important for haemolytic activity and sphingomyelin membrane binding. A P-[W/Y/F]-D motif that is conserved in HALTs and sea anemone, which are known to interact with lipid molecule of membrane are shown in yellow stick. The structure AfFraC with conserved sea anemone aromatic residues are shown in cyan sticks. The dash line circle represents the general lipid-binding region. All the residues numbering are referred to HALT-1. The figures were generated using the program PyMOL (version 2.5.2 Schrödinger, LLC).

The overall structure of rHALT-1 resembles an α-pore-forming toxin (α-PFT) with β sandwich fold consisting of 12 beta strands, two alpha helices, and two 3_10_ helices (Fig. 2A). The 3D structural alignment using the DALI database^30^ revealed that the structure of rHALT-1 shares high similarity to sea anemone actinoporins in particular with AfFraC (Z-score of 22.5, PDB ID: 4TSQ, r.m.s.d 1.9 Å over 160 aligned Cα atoms with 25% sequence identify), AeEqt-II (PDB ID: 1IAZ, Z-score of 22.1 and r.m.s.d 2.0 Å over 158 aligned Cα atoms with 29% sequence identity), AfFraE (PDB ID: 6K2G, Z-score 22.0 and r.m.s.d 1.9 Å over 160 aligned Cα atoms with 25% sequence identity), and ShStn-II (PDB ID: 1GWY, Z-score of 21.8 and r.m.s.d of 1.9 Å over 158 aligned Cα atoms with 27% sequence identity).

The rHALT-1 structure revealed the coordination of all the side chains of aromatic residues including Y90, W107, Y110, W113, F125, Y129, W144, and the P^104^-[W/Y/F]-D^106^ motif, which are highly conserved to its counterparts in sea anemones (Fig. 2B). The equivalent residues of Y110, W113, and Y129 in sea anemone actinoporins were reported to interact with the phosphocholine head group, while P-[W/Y/F]-D motif was found to involve in the binding of sphingomyelin and may play a role in mediating membrane binding^5,7,31,32^. Structural conservation of these aromatic residues and P-[W/Y/F]-D motif suggested that HALT-1 might share a similar functional mechanism to interact with membrane and lipid-binding. Indeed, the substitution of Y110, W113, and Y129 to alanine in rHALT-1 was shown to abolish the haemolytic and cytolytic activity of HALT-1^24,25^ suggested the importance of these aromatic residues in membrane lipid interaction.

HALT-1 protein also consists of *Hydra* specific aromatic residues, which are not present in the sea anemone actinoporins including W25, W33, Y46, F72, W74, Y92, F152, F162, and F166 (Fig. 2B)^23^. Most of these residues (W25, W33, F72, W74, and F152) were found buried and formed hydrophobic interaction or hydrogen bonding with surrounding residues for structural stability of the protein. Nonetheless, residues Y46 and Y92 were shown to be solvent-exposed, but they are located distantly from the lipid-binding aromatic clusters, which indicated that these residues have no direct functional role in lipid-binding (Fig. 2B). There are also aromatic residues that are important for haemolytic activity in sea anemone toxin but not conserved in HALT-1 such as residues L45, L109, and A114. Both L45 and A114 were buried in a hydrophobic environment suggesting their role in structural stability. The L109 equivalent residues in AeEqt-II^W112^, AfFraC^W112^, and ShStn-II^W110^ were shown to have close contact with lipid and membrane suggested their role in membrane binding^5,7^. These differences indicated that HALT-1 might adopt a different membrane interaction compared to those actinoporins.

The crystal structure of rHALT-1 has the residue lysine 76 acetylated to form *N*6-acetyl-l-lysine that interacted with a water molecule through its carbonyl group (Fig. 2A). Previously, neither acetylated lysine nor any post-translational modification was reported for sea anemone actinoporins. As the rHALT-1 was obtained through *E. coli* protein expression system, the acetylation was likely taken place in the bacterium and might have represented an artefact from the protein expression system. Previously, it was shown that HALT-1 mutant with K76A abolished 80–90% of cytolytic and haemolytic activities compared to the wild type^25^. In addition, the mutation of equivalent lysine residue to cysteine (K77C) in AeEqt-II was also shown to have a 100 times reduction in haemolytic activity, and the positive charge of lysine side chain was proposed to be crucial for the function^33^. Acetylation at the ε-amino group of lysyl side-chain would result in the loss of positive charge from the lysine side chain, presumably contributing to the reduced activities of HALT-1. Nonetheless, the rHALT-1 purified from *E. coli* expression system in this study was shown to exhibit haemolytic and cytolytic activities, which suggested that the acetylation at residue K76 does not disturb the function of the toxin^24,25^. To date, it is unknown if the endogenous HALT-1 of *H. magnipapillata* is acetylated as well, hence the functional implication of acetylated K76 residue as compared to ordinary lysine is yet to be investigated.

### Structure comparison of HALT-1 and actinoporins homolog

**Figure 3:**
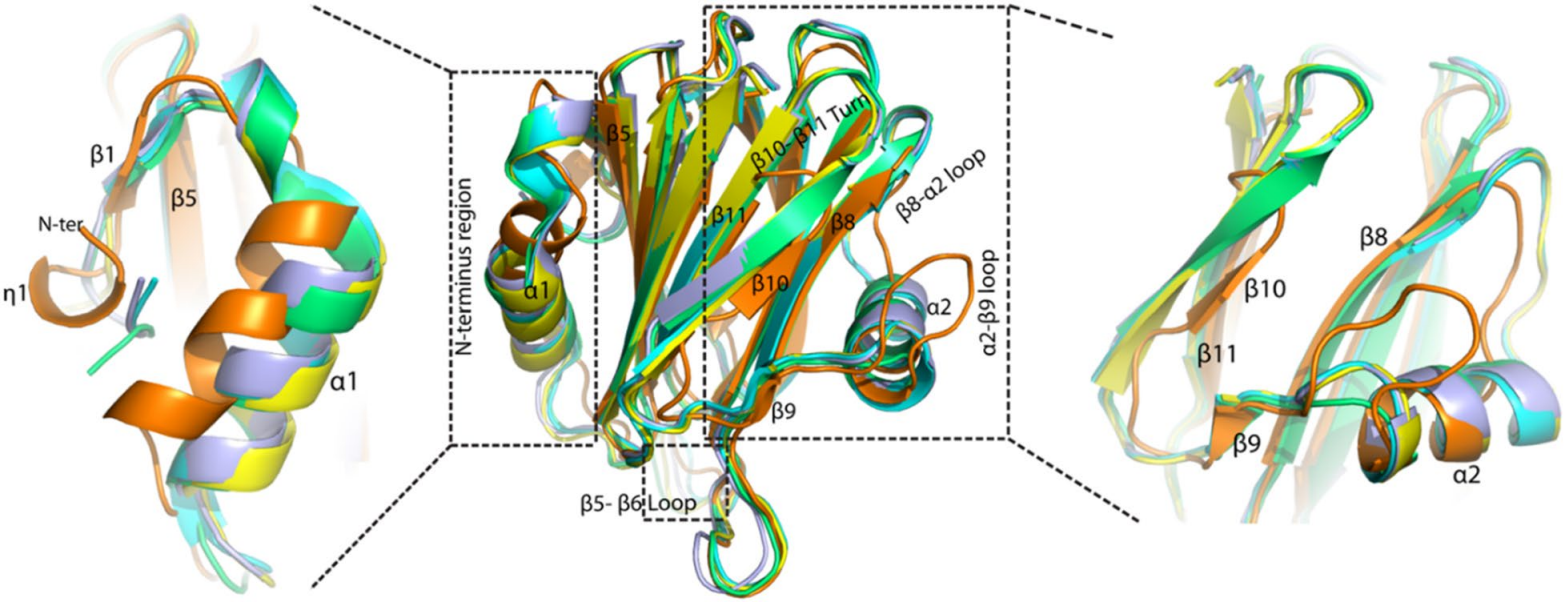
Structure comparison of HALT-1 (orange) with AfFraC (cyan, PDB ID: 4TSQ), AeFraE (blue, PDB ID: 6K2G), AeEqt-II (yellow, PDB ID: 1IAZ), and ShStn-II (green, PDB ID: 1GWY). The regions with high r.m.s deviation of > 4 Å at N-terminal region, β5–β6 loop, β8–α2 loop, and α2–β9 loop are boxed. The overall shorter strands of both β10–β11 and shorter α2 helix of HALT-1 compared to actinoporin homologs are also shown. The figures were generated using the program PyMOL (version 2.5.2 Schrödinger, LLC).

Superimpose of HALT-1 structure with AfFraC, AfFraE, AeEqt-II, and ShStn-II (overall r.m.s.d of ~ 1.9 Å) showed that the biggest r.m.s deviations of > 4 Å were observed at N-terminal region (η1, α1 and η2), β8–α2 loop, β5–β6 loop, α2, α2–β9 loop, and β10–β11 sheets (Fig. 3). All these regions were either fall to or near the deletion or insertion regions of HALT-1 in comparison with actinoporins except β5–β6 loop as shown in Fig. 1. The deviation at β5–β6 loop was due to a missing conserved proline residue found in sea anemone actinoporins in the middle of the HALT-1 loop.

For actinoporins like AfFraC, AfFraE, AeEqt-II, and ShStn-II, an amphipathic N-terminus with ~ 30 amino acids is known to form a long helix that inserts itself into the host membrane as part of the pore formation mechanistic. These ~ 30 N-terminal residues were initially found to have a mixture of β strand and α helix before they went through conformation changes to form the long helix. Similarly, the equivalent amphipathic N-terminal region of HALT-1 was shown to consist of β strand and α helix secondary structures including a 3_10_-helix. Despite the significant deviation at the α1 helix region, the β1 at N-terminus was found to form a sheet with β5 of the β-sandwich core, which was also observed in other actinoporin structures. Hence, these findings suggested that the β1–β5 interaction is a universally conserved feature in regulating the N-terminal conformation change (Fig. 3). The β1 acts as a zip to attach the N-terminal region to the β-sandwich core of the toxin. To form the amphipathic N-terminal helix that penetrates the membrane, a detachment or an ‘unzip’ mechanism that involves β1–β5 interaction is necessary. To date, the lipid-dependent toxin-membrane interaction that can trigger the detachment of β1 from the β-sandwich core domain remains elusive.

The rHALT-1 was found to have a truncated version of β8–α2 loop and a shorter α2 helix, which resulted from the deletion of five amino acids at this region when compared to the actinoporin homologs (Fig. 1). The β10 and β11 strands were also found to have five amino acids deletion, which resulted in the formation of a short version of β10–β11 sheets (Fig. 3). In other actinoporins, these two regions contain an array of basic residues that are conserved among sea anemone actinoporins, and these basic residues make interactions with the negatively charged phospholipid head group. In ShStn-II, the array of basic residues is comprised of K118, K123, R124, R125, K149, R156, and R175. The K118, K123, R124, and R125 residues are positioned in the β7–α2 loop (corresponding to β8–α2 loop in HALT-1) and K149, R156, and R175 residues are positioned in the β8–β10 strands^5^. The HALT-1 has no identical basic residues to the corresponding positions of ShStn-II except for H117 and K122 (equivalent to residue K118 and K123 of ShStn-II). It was suggested that the basic residues in ShStn-II assisted the protein to stably adhere to the cell membrane after the insertion of the N-terminus into the membrane^15^. The lack of basic residues in these two protein regions might contribute to the less cytotoxicity of rHALT-1 to the human cells as compared to other actinoporins^23,25^. In addition, the α2–β9 loop is longer than its counterpart due to a 4–5 residues insertion. These structural differences observed in HALT proteins may contribute to the difference in lipid-binding specificity as compared to the sea anemone actinoporins (see “Lipid-binding model” section below for further details).

**Figure 4:**
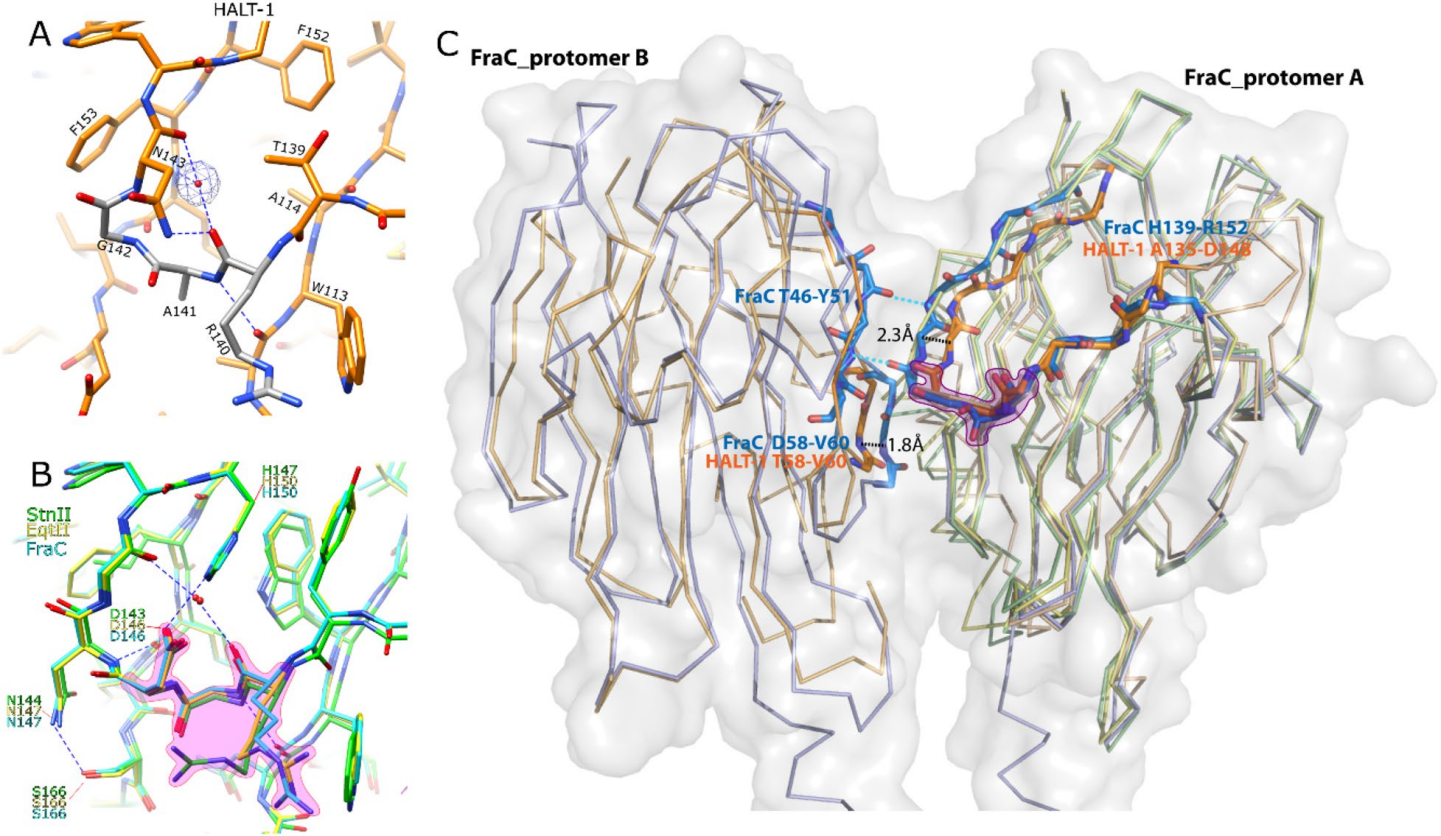
Structural comparison of RAG motif of region HALT-1 (orange) with RGD motif of AfFraC (blue, PDB ID:4TSQ), AeEqt-II (yellow, PDB ID: 1IAZ), and ShStn-II (green, PDB ID: 1GWY). Zoom in views of the RAG motif region in HALT-1 (**A**) and RGD motif region in sea anemone actinoporins (**B**). For HALT-1, the protein residues within the RAG motif are coloured as grey. The 2Fo-Fc maps for the water molecule are contoured at 1.0 σ. (**C**) Superimpose of the protein backbone of HALT-1 onto assembled pore structure of AfFraC (PDB ID:4TSY, blue) showing the protein–protein interaction between β9–β10 and β3 from adjacent protomer. The protein residues within the RGD/RAG motif are highlighted in purple. The shorter β-turn in HALT-1 resulted in G142-W144 (equivalent to N147-W149AfFraC) shifted ~ 2.3 Å toward the β-sandwich core as compared with other sea anemone actinoporins. The residues N147–W149 in AfFraC of one protomer A form an intermolecular β-sheet with residues M48–T50 of another protomer B that is important for oligomerisation. A 1.8 Å shift of T58-V60HALT-1 towards the β-sandwich core compared to D58–V60 AfFraC of protomer B of AfFraC was also observed. The blue dashed lines represent hydrogen bonds with distance ≤ 3 Å. Protein residue is presented as sticks with the nitrogen atoms coloured as dark blue and oxygen atoms coloured as red. The figures (**A**) and (**B**) were generated using the program Chimera^34^ while figure (**C**) were generated using the program PyMOL (version 2.5.2 Schrödinger, LLC).

In sea anemone actinoporins, a highly conserved RGD motif has been proposed in the involvement of protein oligomerisation^27^. However, this motif is not conserved in HALT-1 and it exists as a ^140^RAG^142^ motif that contributed to the formation of a four residues type II β turn between β9 and β10 (Fig. 4A). This turn was stabilised by (1) a weak hydrogen bond between the carbonyl oxygen atom of R140 and amino nitrogen of N143 and (2) a water molecule that hydrogen-bonded with carbonyl oxygen atoms of R140 and N143, as well as amino nitrogen of L154. In contrast, in sea anemone actinoporins, the RGD motifs are highly conserved and found to reassemble a five amino acid residues turn between β9 and β10 (Fig. 4B), which is wider and bigger than HALT-1. Similar to HALT-1, the hydrogen bonding among the main chain atoms in the RGD motif region and a highly coordinated water molecule help to stabilise the turn. In addition, there are additional hydrogen bonds between the D146 and H150 (residue numbering based on AfFraC), which are missing from the HALT-1 (Fig. 4B). The mutation of the RGD motif of ShStn-II into DGR or EAQ resulted in mostly an insoluble protein^27^, indicating that the RGD motif and presumably RAG motif as well are crucial for the toxin stability. Regardless, the arginine residues in both RGD and RAG motifs are in a similar position with their side-chain pointing towards the solvent. An overlay of HALT-1 with sea anemone actinoporins showed that the shorter β-turn in HALT-1 resulted in the main chain atoms of G142–W144 (equivalent to N147–W149^AfFraC^) shifted ~ 2 Å towards the β-sandwich core as compared to other sea anemone actinoporins. In AfFraC, the residues N147–W149 of one protomer (A) form an intermolecular β-sheet with residues M48–T50 of another protomer (B) and this interaction is important for oligomerisation (Fig. 4C). Interestingly, there is also a ~ 2 Å shift of main chain atoms at the region T58–V60^HALT-1^ towards the β-sandwich core as compared to D58–V60 of protomer B of AfFraC. This D58–V60 region is located closer to the centre lumen and next to the N147–W149^A^–M48-T50^B^ intermolecular-β-sheet (Fig. 4C). This observation suggested that the changes of RGD (sea anemone) to RAG motif, together with the structural divergence at T58–V60 region could reduce the interface distance between two protomers of HALT-1 by ~ 2 Å and lead to a different outer pore size of HALT-1 as compared to AfFraC^23^.

Conservation analysis using ConSurf revealed that most of the conserved residues of HALT-1 are found in the lipid-binding site or buried within the β-sheet when compared to its homologs (Supplementary Fig. S3). Most of the solvent-accessible residues found on the surface of the HALT-1 protein are not conserved. We speculate that this could be due to the adaptation of the toxin towards freshwater prey in comparison to the marine environment inhabited by sea anemones. To further investigate this, analysis of the surface electrostatic potential of the HALT-1 and other sea anemone actinoporins was conducted at different salt concentrations. Three salt concentrations were selected to reflect the different ionic strengths of the toxin environments, which were freshwater (≈ 0.01 M), physiological condition (≈ 0.15 M), and seawater (≈ 0.70 M) (Supplementary Fig. S4). Regardless of the salt concentrations, the lipid-binding region remains positively charged, indicating the minimal impact of salt concentrations on the lipid-binding ability of actinoporins as suggested in the previous study^10^. Nonetheless, overall surface charges of HALT-1 at low salt condition were more similar to the surface charges of actinoporin like AeEqt-II and AfFraC in high salt condition (0.15–0.7 M). Interestingly, no significant electrostatic potential changes were observed from freshwater to marine water for ShStn-II. Regardless, we cannot rule out that solvent-exposed residues of HALT-1 may have evolved to adapt to the freshwater environment.

### Lipid-binding model

**Figure 5:**
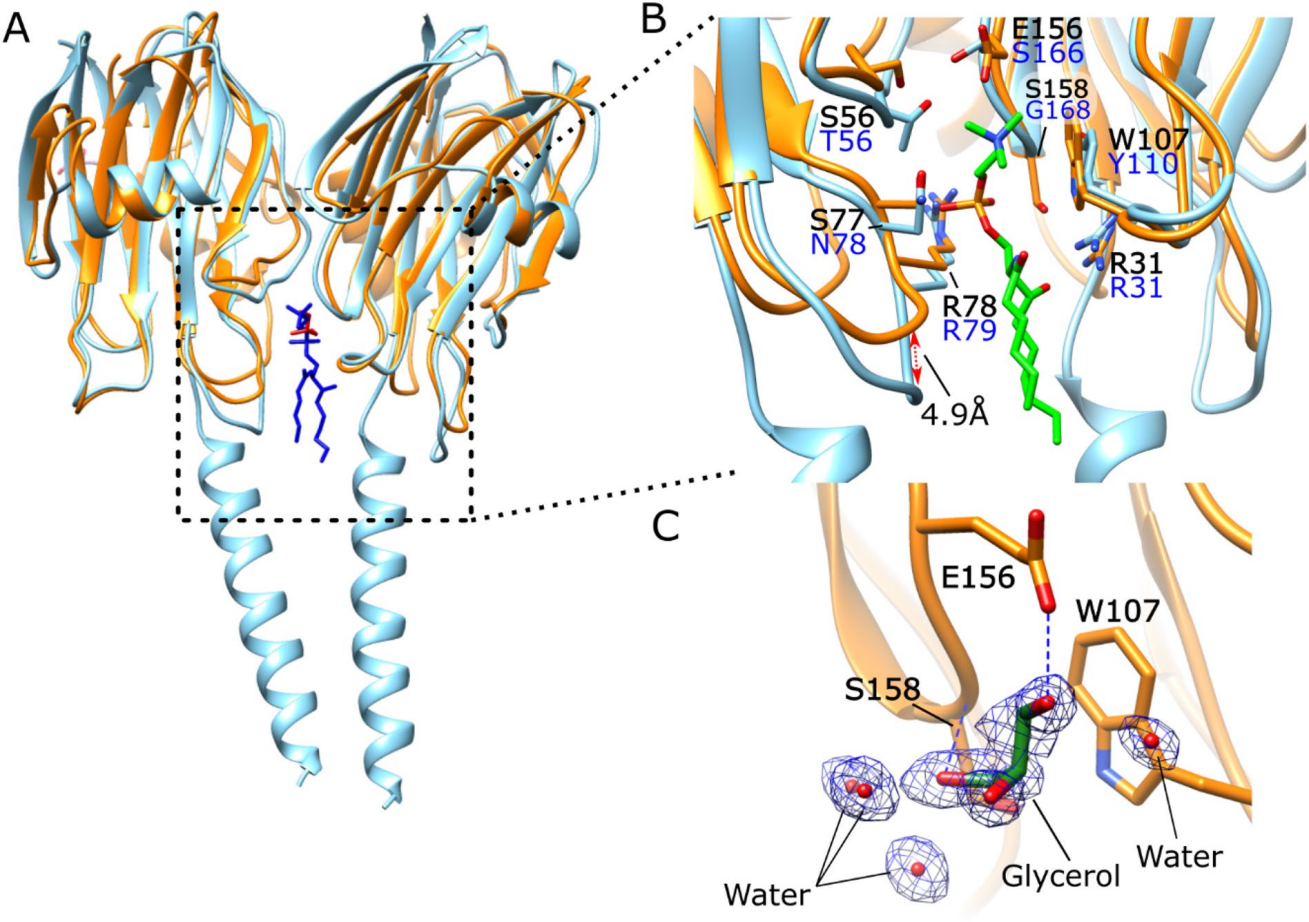
Structural comparison of actinoporin lipid L1 binding mode. (**A**) Superimpose of 2 molecules of HALT-1 (orange) onto two AfFraC protomers bound with lipid L1 position (blue, PDB ID: 4TSY) are shown. DHPC and glycerol molecules are coloured as dark blue and red, respectively. (**B**) Zoom in view showing interaction with DHPC molecule. (**C**) Interaction of HALT-1 with a glycerol molecule. The 2Fo-Fc maps for the glycerol molecule is contoured at 1.0 σ. Residues that interact with the lipid are shown as stick. Putative hydrogen bonds are shown as blue dashed lines. Ligands are depicted as sticks with the carbon, phosphate, nitrogen, and oxygen atoms as green, orange, blue, and red, respectively. Residues for HALT-1 and AfFraC protein are labelled in black and blue, respectively. The figures were generated using the program Chimera^34^.

In the HALT-1 crystal structure, one of the glycerol molecules that formed hydrogen bonding with the side chain of E156 and main chain amide of S158, was found located in the lipid-binding site mimicking the interaction of the lipid L1 within the assembled AfFraC pore structure^7^ (Fig. 5). In the AfFraC pore structure, the lipid L1 was found between actinoporin protomers, contributing to the formation of the transmembrane pore. The superimposition of the HALT-1 onto the assembled actinoporin pore structure revealed that most residues involved in the interaction with lipid L1 were similar and well conserved (Fig. 5B). This finding included a highly conserved residue R31, which is found conserved in all actinoporin sequences (Fig. 1) and responsible for the insertion of the amphipathic N-terminal α-helix into the cellular membrane^35^.

**Figure 6:**
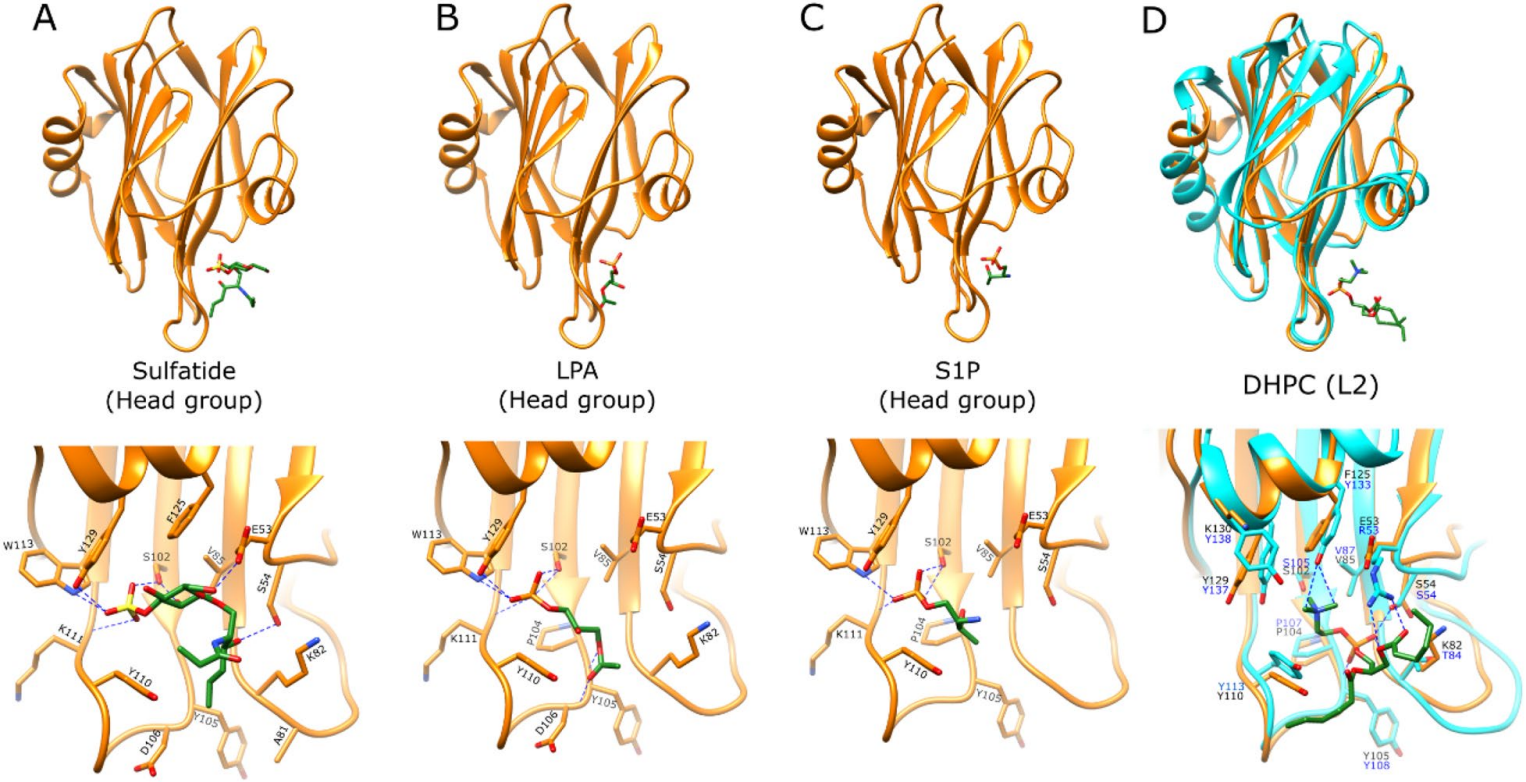
Structural analysis of the lipid-binding region of HALT-1. Head group of (**A**) sulfatide, (**B**) lysophosphatidic acid (LPA), and (**C**) sphingosine-1-phosphate (S1P) are docked onto HALT-1. (**D**) Superimpose of HALT-1 with AfFraC protein bound with DHPC ligand in L2 position (blue, PDB ID: 4TSQ). Ligands are depicted as sticks with the carbon, phosphate, nitrogen, oxygen, and sulphur atoms as green, orange, blue, red, and yellow, respectively. Putative hydrogen bonds are represented by blue dashed lines. The figures were generated using the program Chimera^34^.

The molecular docking was conducted as an attempt to understand the sphingolipids that have been previously shown to bind HALT-1. The head group molecules of sulfatide, lysophosphatidic acid, and sphingosine-1-phosphate were generated and docked into the HALT-1 crystal structure using AutoDock Vina. The lipid chain was not included during the molecular docking due to its floppiness. The best solution for all the head group molecules, as judged from AutoDock Vina’s scoring, was found to be docked into the lipid-binding region, analogously to the binding mode of lipid 2 (L2) pose as seen in AfFraC lipid complex (Fig. 6). Structure comparison of HALT-1 and AfFraC revealed that the L2 pose regions were surrounded by highly conserved residue S102, and conserved aromatic clusters (Y105, Y110, W113, F125, and Y129) (Fig. 6D).

The molecular docking results showed that the sulfate and galactose of the sulfatide head group were possibly forming hydrogen bonding with the side chains of E53, S54, S102, W113, and Y129 as well as the main chain oxygen atom of K111. Similarly, the phosphate group of lysophosphatidic acid (LPA) head group was hydrogen bonded with side chain S102, W113, Y129, and main chain oxygen atom of K111, with additional hydrogen bonding interaction observed between the LPA head group and backbone amide of D106 and Y105. For sphingosine-1-phosphate (S1P) head group, its phosphate group formed hydrogen bonding with the side chain of S102, Y129, W113, and main chain oxygen atom of K111 (Fig. 6). These observations showed that the residues S102, W113, Y129, and K111 of HALT-1 might interact with the sphingolipids with sulfate or phosphate head group.

One of the crystal structures of AfFraC is bound with water-soluble lipid 1,2-dihexanoyl-sn-glycero-3-phosphocholine (DHPC), which shares the same head group as sphingomyelin at the lipid 2 (L2) pose^7^. A comparison between HALT-1 and AfFraC in complex with DHPC revealed that the change of residue Y133^AfFraC^ (a conserved residue among sea anemone actinoporins) to F125^HALT-1^ (a conserved residue among HALTs) caused the loss of C–H⋯O hydrogen bonds between the side chain oxygen atom of tyrosine and the trimethylammonium methyl group of choline, which was a physical state found in AfFraC-sphingomyelin head group interaction (Fig. 6D)^36^. This could also be one of the factors that weaken the binding between sphingomyelin and HALT-1^23,24^.

**Figure 7:**
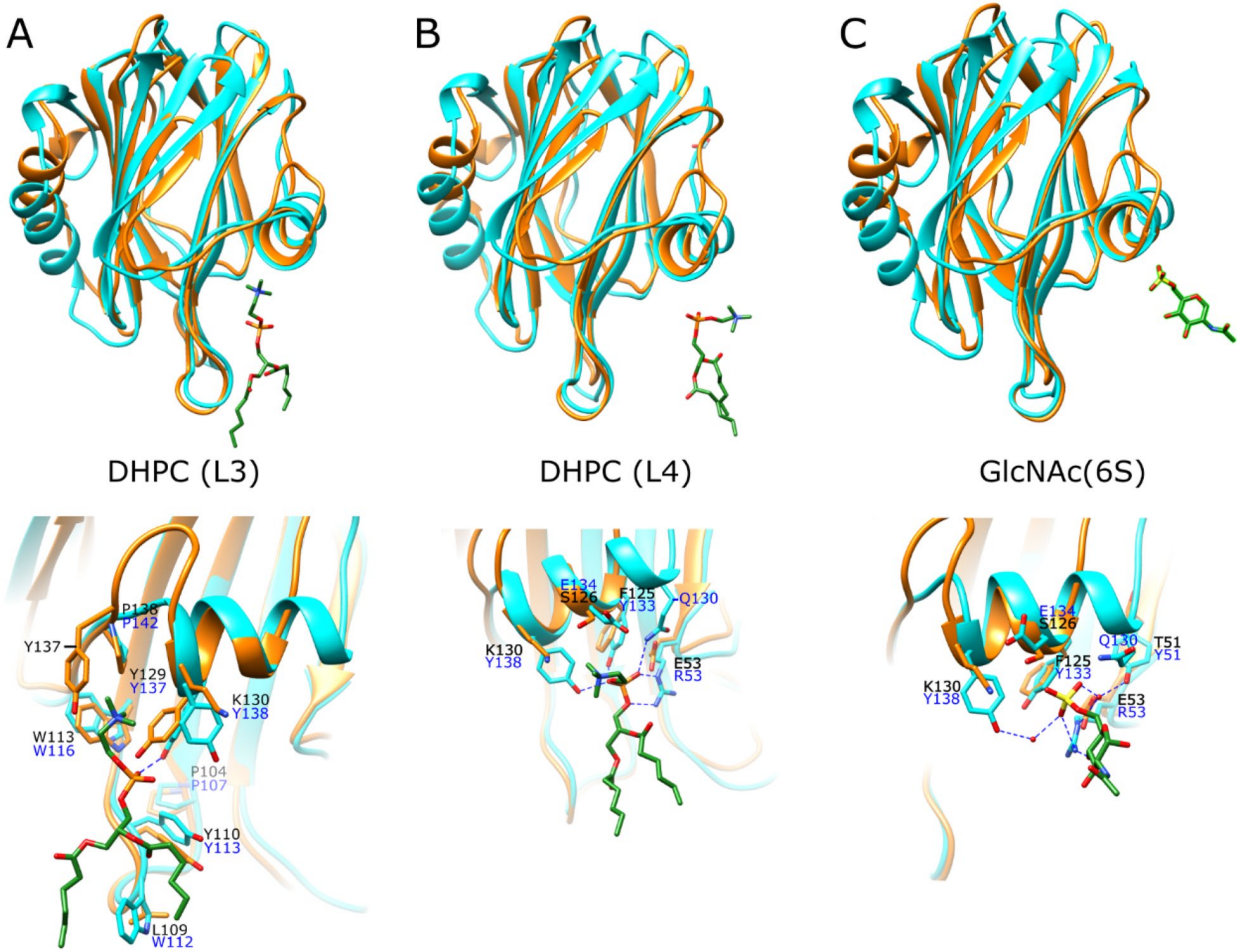
Structural comparison of actinoporin lipid-binding. Superimpose of HALT-1 (orange) with (**A**) AfFraC protein bound with DHPC ligand in L3 position (blue, PDB ID: 4TSO), (**B**) AfFraC protein bound with DHPC ligand in L4 position (blue, PDB:4TSQ), and (**C**) AfFraC protein bound with GlcNAc(6S) (blue, PDB ID:5GWF) are shown. Zoom in view of each binding sites are shown. Putative hydrogen bonds are shown as blue dashed lines. Ligands are depicted as sticks with the carbon, phosphate, nitrogen, and oxygen atoms as green, orange, blue, and red, respectively. Residues for HALT-1 and AfFraC protein are labelled in black and blue, respectively. The figures were generated using the program Chimera^34^.

As none of the docked sulfatide, S1P, and LPA head groups adopted the lipid 3 (L3) pose that was previously shown in AfFraC actinoporin^7^, the HALT-1 was superimposed to the actinoporin structure. Structural comparison of HALT-1 with AfFraC revealed that most of the key residues that coordinates DHPC molecules at lipid L3 binding mode in AfFraC are also conserved in HALT-1 (Fig. 7) with an exception for the W112^AfFraC^, which is replaced by L109^HALT-1^.

Interestingly, none of the docked sulfated galactose head group of sulfatide adopted the lipid 4 (L4) pose, which was previously reported as the binding mode for AfFraC towards sulfated glycan^14^. In AfFraC, the key residues Y51, R53, Q130, Y133, and Y138 are involved in the interaction with DHPC or sulfated glycan molecules in L4 pose, however, they are not conserved in HALT-1 (Fig. 7C). Furthermore, the corresponding Q130 residue found in AfFraC is absent in the HALT-1 due to the deletion of five amino acids region that led to a shorter β8–α2 loop and a truncated helix α2. The possible function of truncated helix α2 in HALT-1 in lipid-binding remains unknown.

Taken together, we present the crystal structure of rHALT-1 and reveal several unique structural features of HALT-1 as compared to the well-studied sea anemone actinoporins. This work provides a new structural insight that lay the groundwork for future experimental designs targeting the specific lipid recognition, membrane binding, and pore formation interfaces of HALTs protein family.

## Methods

### Multiple sequence alignment

Multiple sequence alignment (MSA) of HALT-1 with its paralogs (HALT-2, HALT-3, HALT-4, HALT-5, HALT-6, and HALT-7) and orthologs from *Oulactis orientalis* (OoOr-G_Q5I2B1: DELTA-actitoxin-Oor1b), *Stichodactyla helianthus* (ShStn-I_P81662: DELTA-stichotoxin-She4a and ShStn-II_P07845: DELTA-stichotoxin-She4b), *Heteractis magnific* (HmHmT_P0DMX2: DELTA-stichotoxin-Hmg2b and HmHMg-III_Q9U6X1: DELTA-stichotoxin-Hmg2a), *Heteractis crispa* (HcRTX-SII_P0C1F8: DELTA-stichotoxin-Hcr4b and HcRTX-A_P58691: DELTA-stichotoxin-Hcr4a), *Anthopleura asiatica* (AaBp-1_C5NSL2: DELTA-actitoxin-Aas1a), *Actinia equina* (AeEqt-II_P61914: DELTA-actitoxin-Aeq1a, AeEqt-IV_Q9Y1U9: DELTA-actitoxin-Aeq1c, and AeEqt-V_Q93109: DELTA-actitoxin-Aeq1b), *Actinia fragacea* (AfFraB_MK936900: fragaceatoxin B from AfFraE_MK936901: fragaceatoxin E and AfFraC_B9W5G6: fragaceatoxin C) was conducted using Clustal Omega^37^ and manually refined at region (residues 120–150) according to the structural comparison of HALT-1 and AfFraC. The amino acid numbering of the HALT-1, HALT-2, HALT-5, HALT-6, and HALT-7 sequences was started immediately after the removal of signal peptides^23,24^, while the amino acid numbering of HALT-3 and HALT-4 were referred to the sequences shown in Glasser et al.^23^.

### Expression and purification

HALT-1 coding DNA (504 bp) was previously cloned into pET28a with an MGSSHHHHHHSSGLVPRGSHM affinity tag (6x-His-tag) at the N-terminal of HALT-1 (Supplementary Fig. S1)^24^. The pET28a bearing recombinant HALT-1 (rHALT-1-pET28a) was then transformed into *E. coli* Rosetta Gami (DE3) after the sequence of transcriptional and coding regions were verified by sequencing. For protein production, the *E. coli* was grown in 1 L of Luria–Bertani broth containing 50 µg/mL kanamycin and recombinant HALT-1 (rHALT-1) was expressed by the addition of 1 mM IPTG for 3 h at 37 °C. There were 2 rounds of purification: the recombinant protein was first purified by Ni–NTA affinity chromatography and then eluted in the presence of 20 mM Na_2_HPO_4_ (pH8.0), 300 mM NaCl, and 250 mM imidazole. The volume of eluted protein was reduced to less than 200 µL using the protein concentrator (Amicon 3 kDa, Merck, Darmstadt, Germany) before it was diluted in 1 mL of SEC buffer (25 mM Tris pH 7.5 and 100 mM NaCl). Subsequently, recombinant HALT-1 was purified in the SEC buffer by size exclusion chromatography using HiLoad Superdex 75 PG 16/600 (GE Healthcare, Chicago, IL). Eluted protein was desalted to buffer containing 25 mM Tris pH 7.5 and 50 mM NaCl, then concentrated to a final concentration of 20 mg/mL. The protein concentration was determined using a standard Bradford assay.

### Protein crystallisation and X-ray diffraction data collection

Initial crystallisation screening was carried out with INDEX screen kit (Hampton Research, Aliso Viejo, CA) using the sitting drop vapour diffusion method in 96-well MRC Crystallisation Plates (Molecular Dimensions, Newmarket, UK). The drops containing 0.5 μL rHALT-1 protein (10 mg/mL) and 0.5 μL reservoir solution were equilibrated against 80 μL reservoir solution at 293 K. Crystal hits were identified in several reservoir conditions including F6, F7, F8, F9, G3, G4, G5, H2, H3, H4, H6, H7, and H10. The H6 condition containing 0.2 M sodium formate and 20% w/v polyethylene glycol 3,350 was further optimised. The optimised protein crystals of HALT-1 were obtained after 2 days incubation at 18 °C using the hanging drop vapor diffusion method, in which 1 µL of protein at 10 mg/mL was mixed with 1 µL of reservoir solution containing 0.1 M sodium formate and 22% w/v polyethylene glycol 3350. The crystals were briefly soaked in a cryo-protectant solution containing the growing condition with the addition of 20% v/v glycerol, and then flashed-cooled directly under a 100 K nitrogen stream. A complete data was collected using an in-house X-ray diffractometer Rigaku MicroMax-007 HF (Rigaku, Tokyo, Japan).

### Structure determination, model building, and refinement

X-ray diffraction data of rHALT-1 crystal was integrated, scaled, and merged using iMosflm^38^ and Aimless^39^ in CCP4 program suit. The rHALT-1 structure was solved using the automated molecular replacement webserver Balbes^40^. A solution was found using the model from ShStII (PDB ID: 1GWY, chain A). An initial model was auto-built using ARP/wARP web service with sequence completeness of 87%^41^. The model was further built manually using COOT^42^ and refined using REFMAC5^43^. All the backbone dihedral angles of the residues of rHALT-1 structure fell into the preferred or allowed regions of the Ramachandran plot, as defined by MolProbity analysis^44^. Data collection and refinement statistics are listed in Table 1. Figures were prepared using PyMol (version 2.5.2 Schrödinger, LLC) and UCSF Chimera^34^.

### Molecular docking

The structures of the head group of sulfatide, lysophosphatidic acid (LPA), and sphingosine-1-phosphate (S1P) were drawn and converted to the 3D structure using ChemSketch (Advanced Chemistry Development, Inc). Structure minimisation and partial charges assignment were performed using UCSF Chimera’s “Minimize Structure” and “Dock Prep” features for the ligands model^34^. Autodock Vina 1.1.2^45^ was used for ligand docking with a grid space that covered the entire HALT-1 monomer. The docking results were visualised using UCSF Chimera^34^.

### Structure analysis

The conserved regions in the HALT-1 structure were analysed using ConSurf server^46^. The homolog search was done using BLAST from CLEAN_UNIPROT database^47^. Next, 50 sequences closest to the HALT-1 were then used to calculate the conservation rates. The calculated conservation rates were mapped into the HALT-1 structure and coloured accordingly. To calculate the electrostatic potential, the PDB format files were converted to PQR format with the PDB2PQR server using the PARSE force field and assigned protonation states at pH 7.0^48^. The file was applied to the APBS server by including 0.01 M, 0.15 M, and 0.70 M of ions in the calculation^49,50^.

## Supplementary Information

Supplementary Information.

## Data Availability

The coordinate has been deposited with the Protein Data Bank under accession codes 7EKZ.
