# Supplementary material for: Structural and functional analysis of Hydra Actinoporin-Like Toxin 1 (HALT-1)
Source: Sci Rep. 2021 Oct 19;11:20649. doi: 10.1038/s41598-021-99879-5 (PMC8526580; doi:10.1038/s41598-021-99879-5)
Supplement: Supplementary file 1 — Supplementary Information. [file 41598_2021_99879_MOESM1_ESM.docx]

**Supporting information**


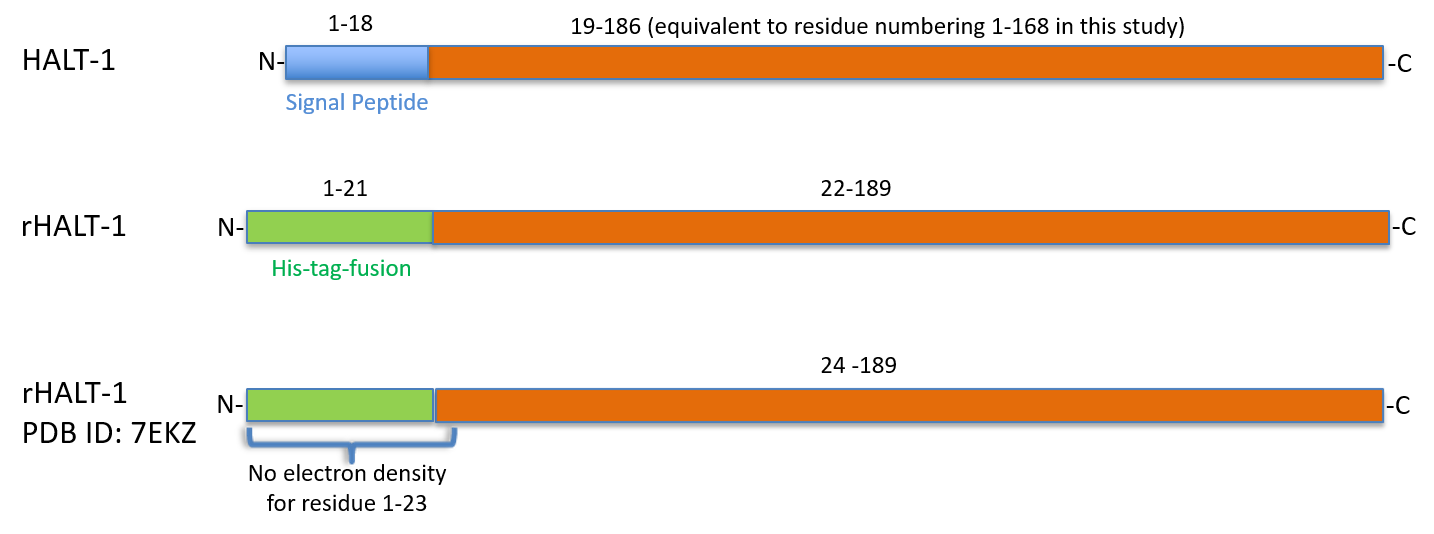


**Figure S1.** The schematic of HALT-1 protein and His-tag-fusion recombinant HALT-1 (rHALT-1).


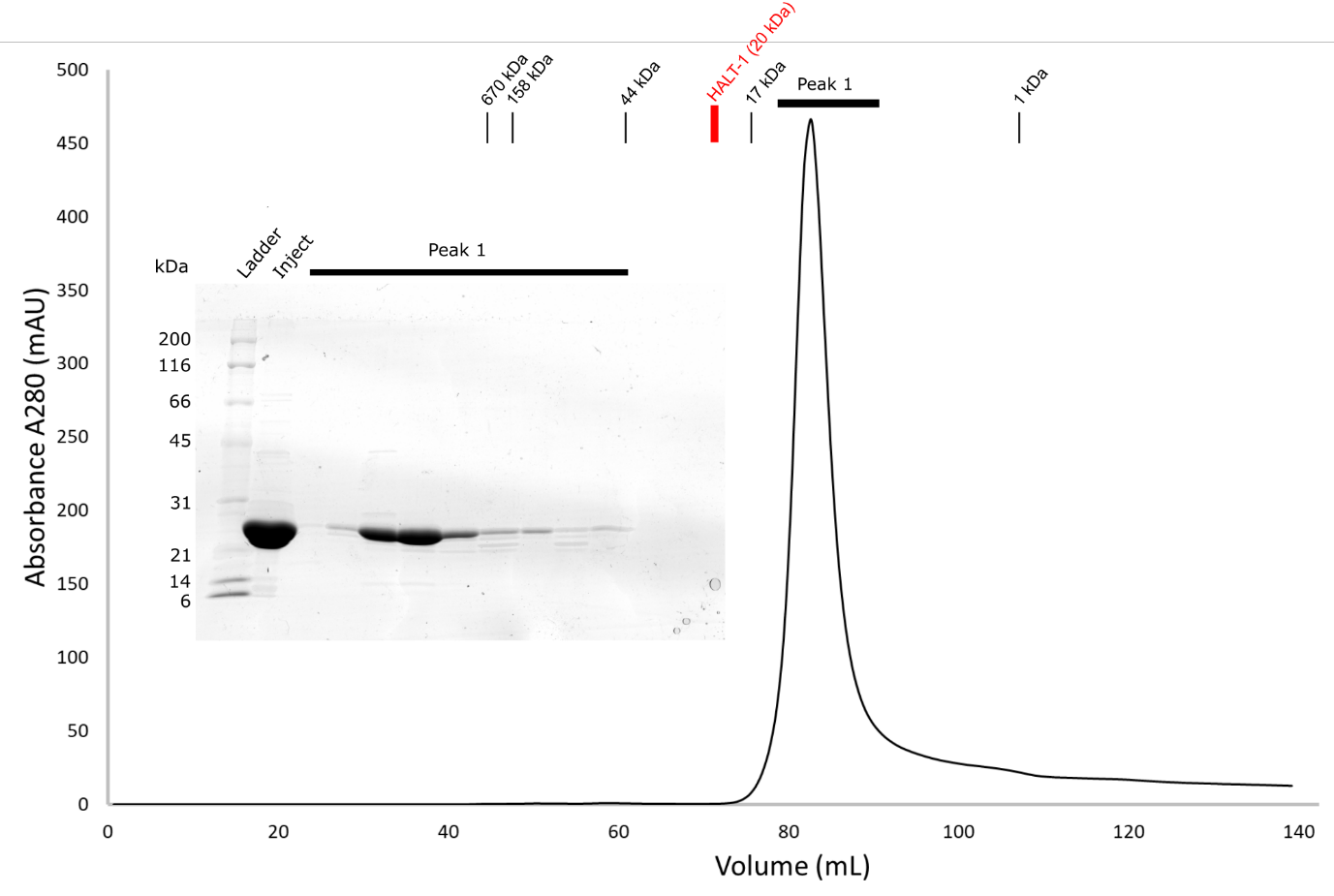


**Figure S2.** rHALT-1 exists as a monomer in solution. rHALT-1 protein was separated using HiLoad Superdex 75 PG 16/600 (GE Healthcare). The elution positions of the protein size standards are indicated at the top. The estimated elution position of the monomer rHALT-1 (20 kDa) based on the protein standards is marked at top as red. An inset image of SDS-PAGE of the eluted peak is shown.


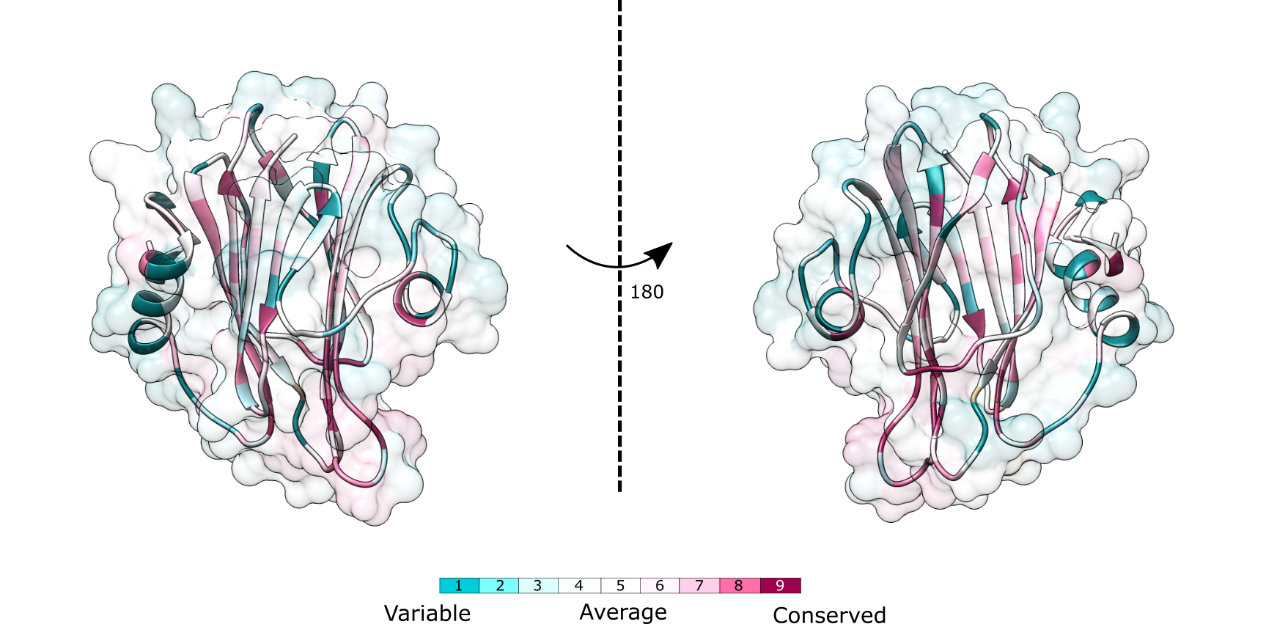


**Figure S3.** Sequence conservation of HALT-1 protein. Each residue was binned from 1 (most variable, blue) to 9 (most conserved, purple) based on residue conservation scores derived from ConSurf^1^ analysis of a sample of 50 HALT-1 homologous sequences and mapped onto the structure of HALT-1. The figures were generated using the program Chimera^2^.


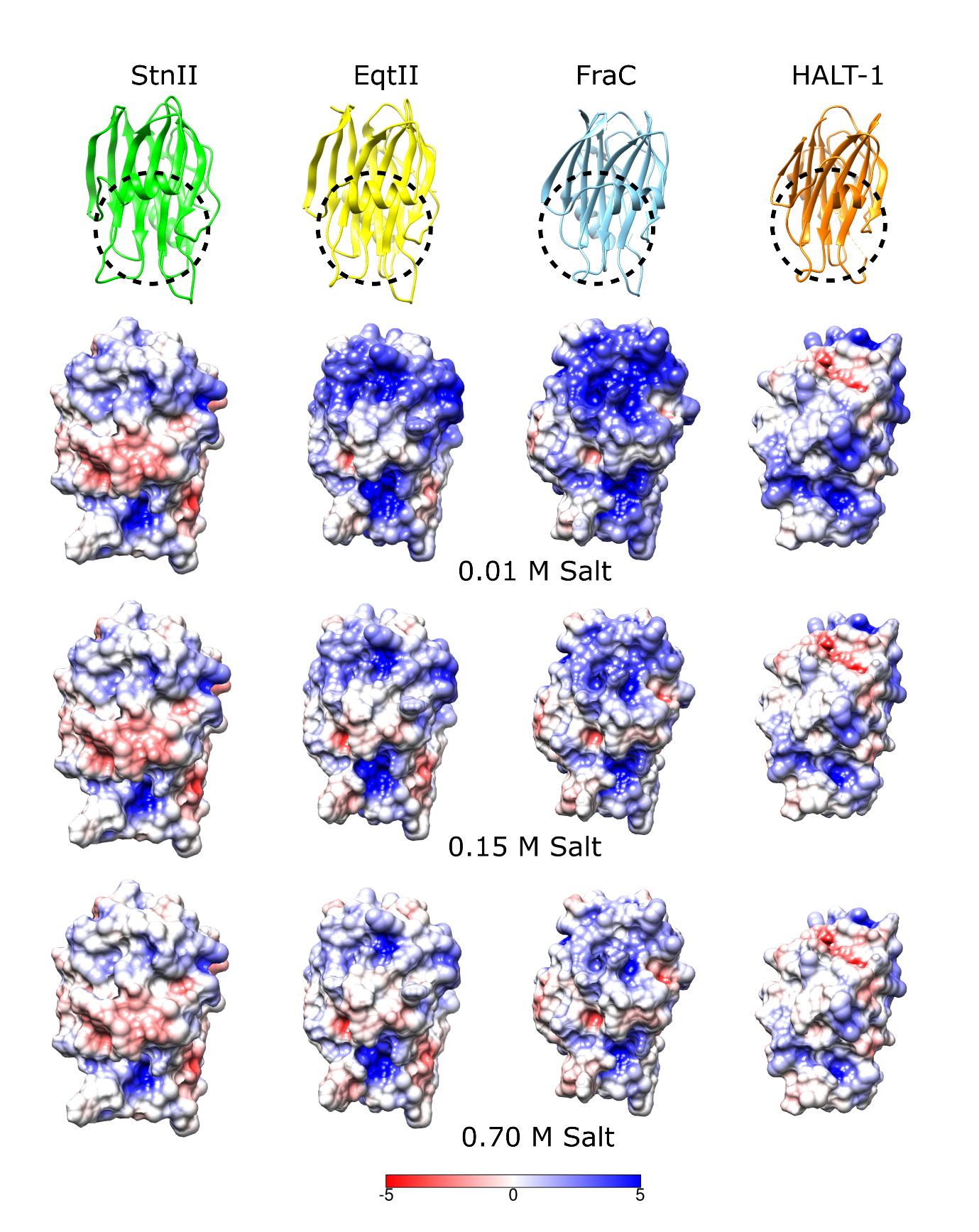


**Figure S4.** Electrostatic potential of HALT-1 in comparison to AfFraC (blue, PDB:4TSQ), AeEqt-II (yellow, PDB: 1IAZ), and ShStn-II (green, PDB: 1GWY) at different salt concentrations. The lipid-binding site is marked by the dashed circle. The electrostatic potential of each protein model at different salt concentrations was calculated using APBS server^3^ and displayed as surface using a colour gradient from -5 (red) to +5 (blue) kT/e. The figures were generated using the program Chimera^2^.

References

1. Ashkenazy, H. *et al.* ConSurf 2016: an improved methodology to estimate and visualize evolutionary conservation in macromolecules. *Nucleic Acids Res.* **44**, W344–W350 (2016).

2. Pettersen, E. F. *et al.* UCSF Chimera-A visualization system for exploratory research and analysis. *J. Comput. Chem.* **25**, 1605–1612 (2004).

3. Jurrus, E. *et al.* Improvements to the APBS biomolecular solvation software suite. *Protein Sci.* **27**, 112–128 (2018).
